# Comparative Analysis of Diversification Rates in Clonal and Non‐Clonal Flowering Plants

**DOI:** 10.1111/ele.70423

**Published:** 2026-06-07

**Authors:** Sonia Kadyan, Jitka Klimešová, Dimitar Dimitrov, Zhiheng Wang, Jan Smyčka, Tomáš Herben

**Affiliations:** ^1^ Department of Botany, Faculty of Science Charles University Praha Czech Republic; ^2^ Institute of Botany Czech Academy of Sciences Průhonice Czech Republic; ^3^ Department of Natural History University Museum of Bergen, University of Bergen Bergen Norway; ^4^ Institute of Ecology and State Key Laboratory for Vegetation Structure, Function and Construction, College of Urban and Environmental Sciences Peking University Beijing China; ^5^ Center for Theoretical Studies Charles University Praha Czech Republic; ^6^ Biological Sciences Simon Fraser University Burnaby British Columbia Canada; ^7^ Biodiversity Research Centre University of British Columbia Vancouver British Columbia Canada

**Keywords:** angiosperms, asexual reproduction, clonality, phylogenetic comparative methods, species diversification, tip rate estimations, trait evolution

## Abstract

Clonality, the process of vegetative reproduction through belowground organs (rhizomes, stolons), occurs in about half of all plant species. It influences key ecological and evolutionary phenomena, including effective population size, meiosis frequency and genet longevity, which may affect diversification rates. This study investigates how clonality impacts diversification in angiosperms by comparing clonal, mixed and non‐clonal genera. Using genus‐level phylogeny and data on clonal status of 16,465 species across 2997 genera, we estimated speciation and net diversification rates for each genus with MoM, DR and BAMM. Our results reveal lower diversification rates in clonal genera in non‐phylogenetic models, consistent with the hypothesis that clonality constrains diversification. This effect weakens when accounting for phylogenetic non‐independence but remains significant overall. We show that monocots show a slightly stronger effect of clonality on diversification than eudicots. Our findings suggest that clonality may limit long‐term diversification in angiosperms, influencing evolutionary dynamics where clonal reproduction predominates.

## Graphical abstract

Using data from 16,465 angiosperm species across 2,997 genera, we classified genera as clonal, mixed, or non‐clonal and estimated diversification using genus‐level phylogeny with DR, MoM, and BAMM. Clonal genera consistently showed lower diversification rates than mixed and non‐clonal genera across all methods. This pattern remained after phylogenetic correction, although the effect was weaker.

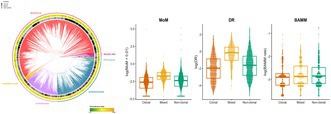

## 1 Introduction

Clonality, the process of asexual reproduction that produces genetically identical offspring from a single parent by vegetative growth, is found across the plant kingdom. This process can occur through different morphological mechanisms, like emerging new ramets along stems, roots or leaves (Klimeš et al. 1997). Notably, clonality is not ubiquitous across all plant types. It has evolved and been lost repeatedly in angiosperms, exhibiting distinct phylogenetic patterns (Herben and Klimešová 2020). This suggests that clonality can be favourable or unfavourable depending on selective pressures. Clonal growth is a widespread and evolutionarily ancient trait in plants, occurring across many lineages and dating back to early vascular plants (Eriksson 1993; Xue et al. 2016). Unlike sexual reproduction, which introduces genetic diversity through recombination and independent assortment, clonal reproduction maintains genetic constancy across generations. Clonality could affect evolutionary dynamics, altering adaptation rates, stability and long‐term species survivability compared to sexual reproduction (Otto and Lenormand 2002; Hadany and Otto 2016). Given its potential implications for evolutionary processes, it is essential to explore how clonality affects patterns of plant diversification.

**FIGURE 1:**
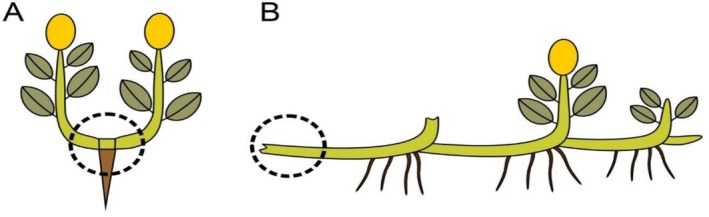
Schematic representation of Non‐Clonal plants (A) and Clonal plants (B). The dotted circle indicates the spot where the seed has germinated, giving rise to the new genetic individual.

As clonality is known to affect the genetic structure and its rate of change, it is plausible to hypothesize that it may also influence broader patterns of diversification. Diversification, defined in macroevolutionary terms as the net balance between speciation and extinction rates, determines the accumulation of lineage diversity over time (Losos and Ricklefs 2009; Morlon 2014). In non‐clonal species (Figure 1A), recombination reshuffles genetic material and promotes genetic diversity. In contrast, clonal species (Figure 1B), which produce genetically identical offspring in addition to sexual ones, can reduce genetic variation within populations (Lynch 1984; Hartfield and Glémin 2016). These differences can be hypothesized to influence speciation in plant lineages, thus affecting overall patterns of diversification, while extinction remains difficult to assess from existing phylogenetic data. Understanding the broader implications of clonality on diversification can bridge the knowledge gap on its influence on evolutionary patterns, and the long‐term species richness dynamics in individual lineages (Ingram and Mahler 2013; O'Meara 2012; Rabosky 2014).

Clonality may affect speciation via several underlying mechanisms. First, clonal plants (Figure 1B) reproduce not only sexually but also asexually (see e.g., Boedeltje et al. 2007; Herben et al. 2014; Vallejo‐Marín et al. 2010), resulting in fewer meiotic events compared to sexually reproducing plants, leading to fewer opportunities for genetic recombination, thereby affecting the generation of genetic diversity. Second, clonal plants (Figure 1B) often have longer lifespans for individual genets (genetic individuals; see Eriksson 1993, de Witte & Stöcklin 2010, see also Ehrlén and Lehtilä 2002), which can stabilize genetic diversity over time but may also reduce the rate at which new genetic variations enter the population. Additionally, the long lifespan of clonal plants (Figure 1B) allows somatic mutations to accumulate over time, which can further influence genetic diversity, especially if the mutations happen in parts of the plant that make new individuals (Lanfear et al. 2010). Together, these factors—frequency of meiosis, clone lifespan and their interactions may yield unpredictable impacts on diversification rates (speciation), potentially resulting in both deceleration and acceleration in clonal plants (Figure 1B). The role of clonality in the diversification of flowering plants has not been studied, leaving significant gaps in our understanding of how it affects macroevolutionary patterns. The key question is whether the long‐term evolutionary trajectories of clonal lineages are more likely to reach a point where further evolution is limited or whether they can persist and continue to diversify (Silvertown 2008; Aarsen 2008).

Here, we address these knowledge gaps by examining how clonality impacts diversification. Our primary goal was to explore the correlation between clonality and diversification using tip diversification rates. We compiled an extensive dataset on clonality and integrated it with the genus‐level phylogeny from Dimitrov et al. (2023), which represents a comprehensive time‐calibrated phylogeny available for angiosperm genera. Using this combined dataset, we assessed speciation (as a proxy for diversification) using three complementary metrics (MoM, DR metric and BAMM), each operating under different assumptions regarding rate estimation and being differently sensitive to different phylogenetic scales. We applied both linear models and phylogenetic generalized least squares (PGLS) to compare diversification rates among strictly clonal, mixed and non‐clonal genera while accounting for phylogenetic non‐independence. To test whether this relationship varies among major lineages, we conducted separate analyses for monocots and eudicots, as these groups are known to show different patterns of morphological constraints for clonality (Klimešová et al. 2018). We examined the following two hypotheses: (H1) clonality is associated with lower diversification rates due to reduced genetic recombination and increased lineage persistence; (H2) the impact of clonality on diversification differs between monocots and eudicots, with a stronger relationship anticipated in monocots, since clonality is more morphologically variable in monocots.

## 2 Methods

### 2.1 Clonality Dataset Assembly

We sourced data from established databases of clonal growth forms. A large portion of our data comes from CLO‐PLA (Klimešová et al. 2017), which originally included 2909 taxa from Central Europe. We also incorporated unpublished data from one of the authors (J.K.), complementing the existing CLO‐PLA dataset with an additional 1686 taxa. These records reflect past fieldwork projects conducted by J.K. and her collaborators across multiple regions (see the Table S1). Additionally, we compiled a clonality dataset from other databases in addition to the CLO‐PLA database, namely from Zhang et al. (2017), Howard et al. (2020), Pausas et al. (2018) and Ülgen and Tavşanoğlu (2024) (Table S1). We used all published sources with reliable clonality data, and a total of 18,003 clonal and non‐clonal species records were aggregated from these sources, representing 10,239 non‐clonal species and 7361 clonal species across 2997 genera. We used the classification of species into clonal/non‐clonal categories from the original studies. It is also important to note that the definition of clonality as used here (Klimešová and de Bello 2009) does not account for clonal reproduction through apomixis and similar seed‐based mechanisms, due to the general rarity of this reproduction mode in our dataset and to the incomplete coverage of our taxa by this information. We then used the clonality data on individual species to classify all genera with at least one species with clonality known into three categories: (i) strictly clonal genera (*n* = 1008), that is, with only clonal species, (ii) ‘mixed’ genera (*n* = 448), including both clonal and non‐clonal species and (iii) strictly non‐clonal genera (*n* = 1541) including only non‐clonal species.

### 2.2 Phylogenetic Tree

All analyses were conducted using the time‐calibrated genus‐level molecular‐informed consensus phylogeny from Dimitrov et al. 2023 with dating constraints 140–210 Ma. A portion of our analyses was also rerun on phylogenies with alternative dating constraints (149–259 Ma and 140–150 Ma), with negligible effect on results (see below). For each genus in the tree, we matched the total number of accepted species it contains according to World Flora online (WFO 2023), which was required for the calculation of diversification metrics (MoM, DR and BAMM) considering the total diversity of angiosperms. Further, our dataset was split into monocots and eudicots based on the identification of their most recent common ancestors.

### 2.3 Taxonomic Harmonization

To correctly match genus names in the clonality dataset with the genus‐level phylogeny, we harmonized our data using World Flora Online (WFO) through the R package World Flora version 1.14–1 (World Flora Online 2023). This process involved aligning our clonal dataset with WFO standards, resolving synonyms to currently accepted names, removing duplicates and correcting taxonomic errors. Only genera present in both the clonality datasets, and the phylogeny were retained for analysis. After matching and filtering, the working dataset comprised 16,465 species (10,028 non‐clonal and 6437 clonal) distributed across the 2997 genera present in the phylogeny of Dimitrov et al. (2023). In case of conflicting information on clonality from different source datasets, we conservatively classified the species as clonal.

### 2.4 Diversification Rate Estimation

We calculated tip‐rate estimates based on a comprehensive angiosperm genus‐level phylogeny (Dimitrov et al. 2023; see Figure S1 for the analytical workflow) using three different methods for estimating diversification rates for the genera: Method of Moments (MoM, Magallón and Sanderson 2001), DR metric (Jetz et al. 2012) and tip estimates of net diversification rate from BAMM (Rabosky and Goldberg 2015). These methods were selected because they span two key gradients of assumptions underlying the estimation of cladogenetic rates, and their combination thus provides a more nuanced view of diversification dynamics across our dataset.

Firstly, all three methods differ in their assumptions about extinction rate. MoM estimates net diversification rate (speciation minus extinction rate), while DR was originally developed as a measure of speciation rate (Jetz et al. 2012) although it has frequently been misinterpreted as a measure of net diversification (pers. comm. Arne Mooers, author of the DR metric). Compared to these two methods, BAMM explicitly estimates speciation and extinction rates for terminal leaves of the phylogeny, allowing to work with both speciation and net diversification. In practice, however, the extinction estimates from BAMM using extant phylogenies are almost agnostic to the true extinction process (Rabosky 2014; Title and Rabosky 2019; Ridder et al. 2025), resulting in most of the signal in net diversification rates reflecting speciation rather than extinction dynamics. Accordingly, we interpret all three metrics primarily as reflecting speciation dynamics, while acknowledging that differences between MoM or BAMM and DR may partly reflect extinction assumptions. In the further text, we are thus using the terms ‘speciation’ and ‘diversification’ interchangeably.

A more substantive distinction among metrics concerns the depth of cladogenetic processes they capture. MoM is calculated as *log(n)/t*, where *n* is the number of species within a genus and *t* is a terminal branch length of the genus on the genus‐level phylogeny. This estimator is agnostic to the deeper branching structures of the phylogenetic tree than the node determining the age of each genus and thus mostly reflects the shallow diversification dynamics at the level of genera. In comparison, the DR metric is an inverted value of equal‐split evolutionary distinctiveness. It thus measures the lengths of the branches leading to every taxon weighted progressively across the tree, with importance exponentially decaying from the tips of the phylogenies toward the deeper branchings. BAMM also models diversification rates across the whole phylogeny, but compared to DR, it regards phylogeny as being generated by a series of relatively rare rate shifts. This assumption makes BAMM estimates fairly conservative, as the taxa covered by each rate shift have very similar estimates of diversification rates (Moore et al. 2016; Ronquist et al. 2021; Ridder et al. 2025). As a result, BAMM estimates are more strongly influenced by deeper phylogenetic structure than MoM or DR.

While MoM is directly defined as a measure of diversification of multi‐species taxa, DR and BAMM are both defined for species‐level phylogenies. We thus had to augment our genus‐level phylogeny to reflect the known within‐genus diversities. For DR metric estimation, we simulated the species‐level branching patterns within each genus using the known diversity of each genus and Yule process (Rangel et al. 2015; Smyčka et al. 2017). We resolved the within‐genus part of the tree of the consensus tree from Dimitrov et al. 2023, 1000 times using the function ‘genus.to.species.tree()’ from R package ‘phytools’ (version 2.5; Revell 2024), custom‐optimized for better performance on very large phylogenies (328,883 species). Subsequently, we calculated DR values for all the 1000 augmented phylogenies using ‘evol_distinct()’ from R package ‘phyloregions’ (version 1.0.9; Daru et al. 2020), also optimized for very large phylogenetic trees. We averaged these species‐level DR estimates across all augmented trees for each genus. However, we also provide here a sensitivity analysis across all the 1000 trees separately (see Table S2). All analyses were conducted in R v.4.2.2 using the packages ape (Paradis and Schliep 2019), phytools (Revell 2012) and caper (Orme et al. 2018).

For BAMM, we used genus level estimates from Dimitrov et al. (2023). The species counts for every genus were used to define the sampling fraction of each genus in BAMM. The resulting tip rate estimates thus reflect the within‐genus diversities along a similar logic depicted for DR in the previous paragraph. Dimitrov et al. provide BAMM estimates for three different sets of dating constraints: the first encompasses a broader temporal interval (min = 149 Ma, max = 256 Ma) to cover commonly accepted average ages, the second employed a median estimate (min = 140 Ma, max = 210 Ma), and the third focused on the highest probabi(Revell 2012) and caper (Orme et al. 2018).

For BAMM, we used genus level estimates from Dimitrov et al. (2023). The species counts for every genus were used to define the sampling fraction of each genus in BAMM. The resulting tip rate estimates thus reflect the within‐genus diversities along a similar logic depicted for DR in the previous paragraph. Dimitrov et al. provide BAMM estimates for three different sets of dating constraints: the first encompasses a broader temporal interval (min = 149 Ma, max = 256 Ma) to cover commonly accepted average ages, the second employed a median estimate (min = 140 Ma, max = 210 Ma), and the third focused on the highest probability density around an average age of approximately 145 Ma (min = 140 Ma, max = 150 Ma). While the older dating constraints generally result in lower tip rate estimates in absolute values, all three sets of estimates remain an almost perfect linear transformation of each other (*R* ^2^ > 0.95). As we were interested in the relative comparison of diversification rates between clonal and non‐clonal species, rather than the absolute values of diversification rates, we used estimates for the constraint 140–210 Ma and provided the results for the other two constraints in the (Figure S2).

### 2.5 Statistical Analyses

We used linear models with the three genus‐level diversification rates (MoM, DR and BAMM) as response variables and genus clonality categories (strictly clonal, mixed and strictly non‐clonal) as a predictor. We also used the (log‐transformed) number of species with clonality data available as a covariable to account for the statistical effect that the better sampled genera and genera with larger total within‐genus branch length are more likely to include both clonality types. All the response variables were log‐transformed before analysis. While there is no a priori mechanistic reason for transformation of diversification rates, all three of them have shown strongly positively skewed distributions both in raw data and in model residuals that could be efficiently normalized by logarithmic transformation. This positive skew is very common in empirical extant phylogenies in general and reflects tree imbalance (Blum and François 2006). We compared the models with clonality predictors with a model without clonality by the Akaike Information Criterion (ΔAIC), where ΔAIC > 2 to test if the inclusion of clonality category indicates a meaningful improvement in model fit. In all our models, we used contrast coefficients of clonal versus mixed and clonal versus non‐clonal as measures of the strength of the effect of clonality on diversification.

The tip diversification rates are phylogenetically autocorrelated, that is, closely related tips have more similar values of diversification rates. However, this autocorrelation cannot be straightforwardly corrected using techniques designed for evolution of traits, because the nature of rate evolution along the branches is more complex and depends on the mathematical properties of particular diversification metrics. As an extreme example, the DR metric of two sister species is identical by definition regardless of their terminal branch length, which cannot be captured by a Brownian model of trait evolution or any of its commonly used transformations. Both phylogenetically corrected and uncorrected results are commonly used in literature (see e.g., Jetz et al. 2012; Rabosky et al. 2018; Smyčka et al. 2023). We resolved this issue by presenting results of models both with and without phylogenetic autocorrelation components, emphasizing that the phylogenetically corrected results represent the (probably overly) conservative choice. To do so, we used a phylogenetic generalized least squares model with lambda transformation as implemented in ‘phylolm’ package (version 2.6; Ho and Ané 2014) in R.

To explore the differences between monocots and eudicots, we conducted interaction tests. We fitted two models, one (i) with the main effect of clonality, the main effect of lineage (monocot vs. eudicot) and log‐transformed sampling effort (number of species assessed for clonality) as a covariate, and a second model (ii) that also included the interaction between clonality and lineage, and compared them using AIC. Further, we fitted models with clonality and sampling effort for each lineage separately.

To account for the uncertainty in calculating DR based on unresolved species‐within‐genus relationships, we conducted a sensitivity analysis using 1000 randomly generated trees with infrageneric phylogenies, based on the Dimitrov et al. (2023) consensus tree and data on species numbers per genus. For each of these trees, we again fitted two models (Aarsen 2008): a linear model (LM) without phylogenetic correction and (Beaulieu and O'Meara 2016): a phylogenetic generalized least squares (PGLS) model that accounts for evolutionary non‐independence among species, assessing the robustness of our inference on DR values by calculating 95% confidence intervals of both the PGLS and LM parameters across 1000 randomly generated trees used to calculate the average DR. These intervals were extremely narrow, indicating that uncertainty had a negligible impact on the model outcomes. (Table S2).

## 3 Results

**FIGURE 2:**
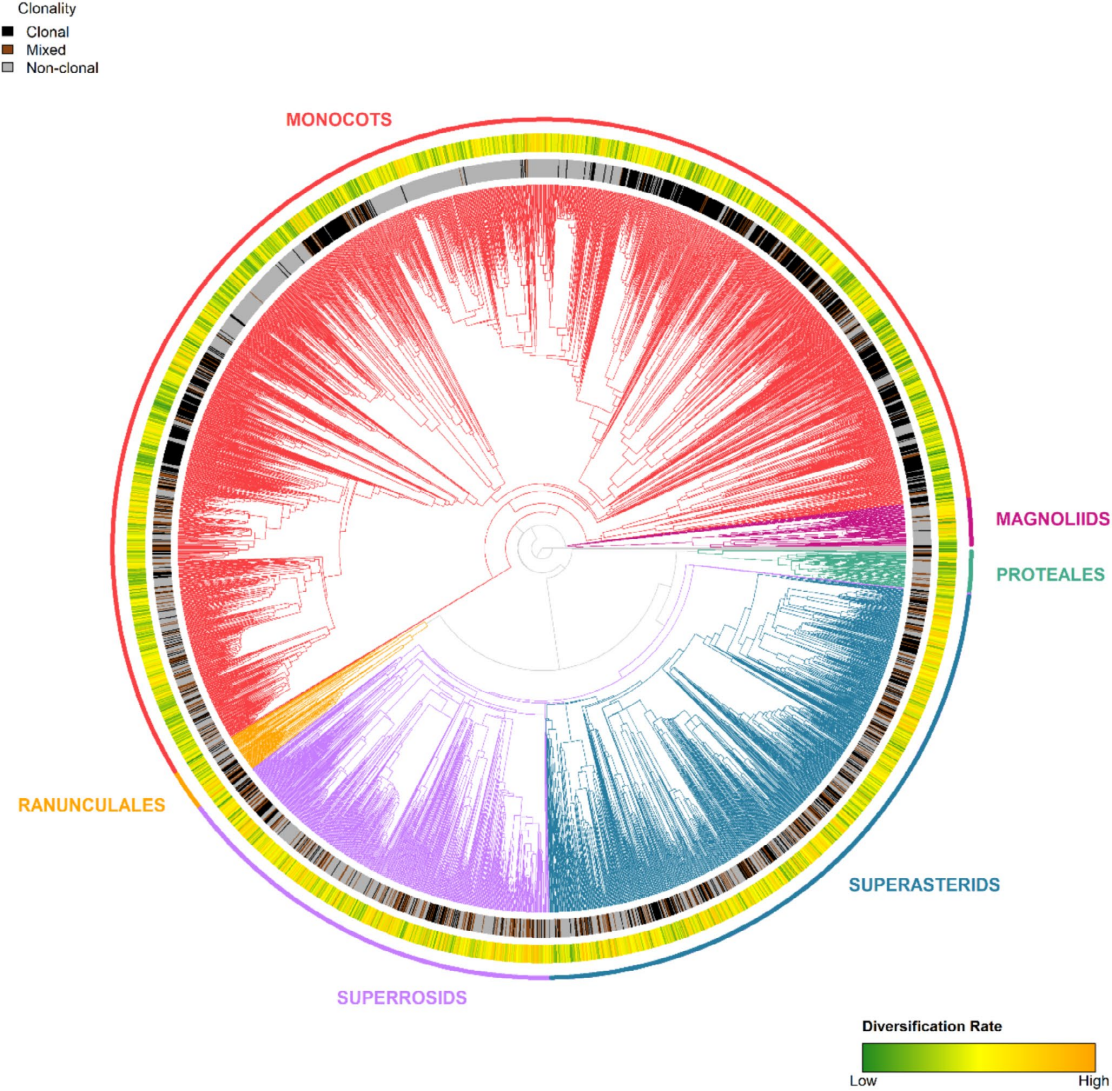
Phylogenetic distribution of clonality and diversification rates across angiosperm genera. The circular phylogeny displays genera with branch colours indicating major clades: Magnoliids (pink), Ranunculales (orange), monocots (red), Proteales (teal), super‐rosids (purple) and super‐asterids (blue). The inner ring indicates clonality status (black = clonal genera; grey = non‐clonal genera; brown = mixed genera). The outer ring shows tip‐level diversification rates (DR; Jetz et al. 2012) on a log scale from low (green) to high (orange).

Clonality exhibits a non‐random distribution across the phylogenetic tree (Figure 2), with clonal species clustered within specific lineages. Several major clades, notably monocots and super‐asterids, show high concentrations of clonal taxa, whereas other lineages contain comparatively few clonal species.

### 3.1 Effects of Clonality on Diversification Rates Across All Angiosperms

We analysed 2997 genera with known clonal status, out of the total 14,244 genera in the Dimitrov et al. (2023) phylogeny. Across all angiosperms, strictly clonal genera exhibited lower diversification rates compared to both mixed and non‐clonal genera (Figure 2).

**FIGURE 3:**
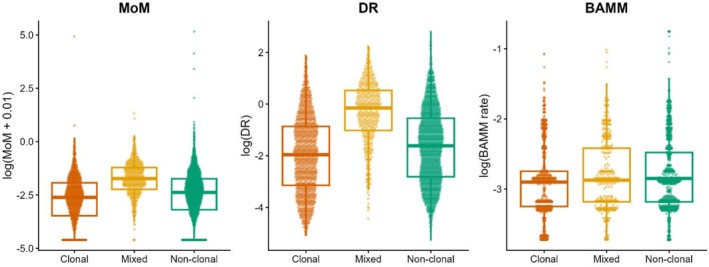
Diversification rates in clonal, mixed and non‐clonal lineages. Log‐transformed diversification rate estimates inferred using Method‐of‐Moments (MoM), the DR statistic and BAMM. Boxes show interquartile ranges with medians; whiskers extend to 1.5 × IQR; points represent individual genera. Mixed genera show higher diversification rates partly due to the effect of sampling effort. In our models, this was corrected for by including the sampling effort as a covariate. In the BAMM panel, horizontal clustering of points reflects shared rate regimes assigned to multiple lineages, consistent with the method's conservative estimation of diversification‐rate variation.

For MoM diversification rates, PGLS analysis showed strong support for an effect of clonality (ΔAIC = 47.88; *λ* = 0.51), with both mixed (*β* = 0.303, *p* < 0.001) and non‐clonal genera (*β* = 0.500, *p* < 0.001) exhibiting significantly higher diversification rates than strictly clonal genera (Table 1, Figure 3). LM similarly supported inclusion of clonality (ΔAIC = 29.15), with elevated diversification in mixed (*β* = 0.315, *p* < 0.001) and non‐clonal genera (*β* = 0.216, *p* < 0.001).

**TABLE 1:** Effects of clonality on diversification rates across all angiosperms.

Response	Model	*N*	Intercept (Clonal)	Clonal vs. Mixed	Clonal vs. Non‐clonal	ΔAIC	*λ*
MoM	LM	2997	0.469***	0.315***	0.216***	29.15	
	PGLS	2997	0.738***	0.500***	0.303***	47.88	0.51
DR	LM	2997	0.044***	0.053	0.107***	22.22	
	PGLS	2997	0.512***	0.097	0.099*	0.4	0.75
BAMM	LM	2997	0.805***	0.187*	0.133*	4.52	
	PGLS	2997	0.007*	−0.001	−0.001	−3.94	1

*Note:* This table summarizes the results of linear models (LM) and phylogenetic generalized least squares (PGLS) examining the effect of clonality on log‐transformed diversification rates across 2997 angiosperm genera. Response variables are: BAMM, net diversification rate from Bayesian Analysis of Macroevolutionary Mixtures; DR, tip‐level diversification rate; MoM, Method‐of‐Moments diversification rate. The models report the intercept (baseline for clonal species) and contrasts comparing clonal species to mixed and non‐clonal species. ΔAIC, AIC of the model without clonality—AIC of the model including clonality (positive values indicate support for inclusion of clonality). *λ* = Pagel's *λ* (phylogenetic signal) for PGLS models. Significant coefficients are indicated as ***p 〈 0.001; **p 〈 0.01; *p 〈 0.05.

For DR diversification rates, PGLS showed weak support for clonality (ΔAIC = 0.40; *λ* = 0.75), with only non‐clonal genera showing significantly higher diversification than clonal genera (*β* = 0.099, *p* < 0.05), while mixed genera did not differ significantly (*β* = 0.097, *p* > 0.05). LM showed stronger support (ΔAIC = 22.22), with non‐clonal genera exhibiting higher diversification (*β* = 0.107, *p* < 0.001), but mixed genera again showing no significant difference (*β* = 0.053, *p* > 0.05).

For BAMM net diversification rates, PGLS showed no support for clonality (ΔAIC = −3.94; *λ* = 1.0), with neither mixed (*β* = −0.001, *p* > 0.05) nor non‐clonal genera (*β* = −0.001, *p* > 0.05) differing from clonal genera. LM showed weak support (ΔAIC = 4.52), with mixed (*β* = 0.187, *p* < 0.05) and non‐clonal genera (*β* = 0.133, *p* < 0.05) exhibiting slightly higher diversification. The complete phylogenetic signal (*λ* = 1.0) suggests BAMM estimates of diversification rates carry a strong phylogenetic signal that cannot be unambiguously attributed to clonality or species relatedness. The lack of clonality signal in PGLS thus likely reflects the nature of BAMM, which assumes few evolutionary rate shifts and has been shown to produce overly conservative estimates (Moore et al. 2016; Ronquist et al. 2021; Ridder et al. 2025).

### 3.2 Clade‐Specific Effects of Clonality on Diversification

**FIGURE 4:**
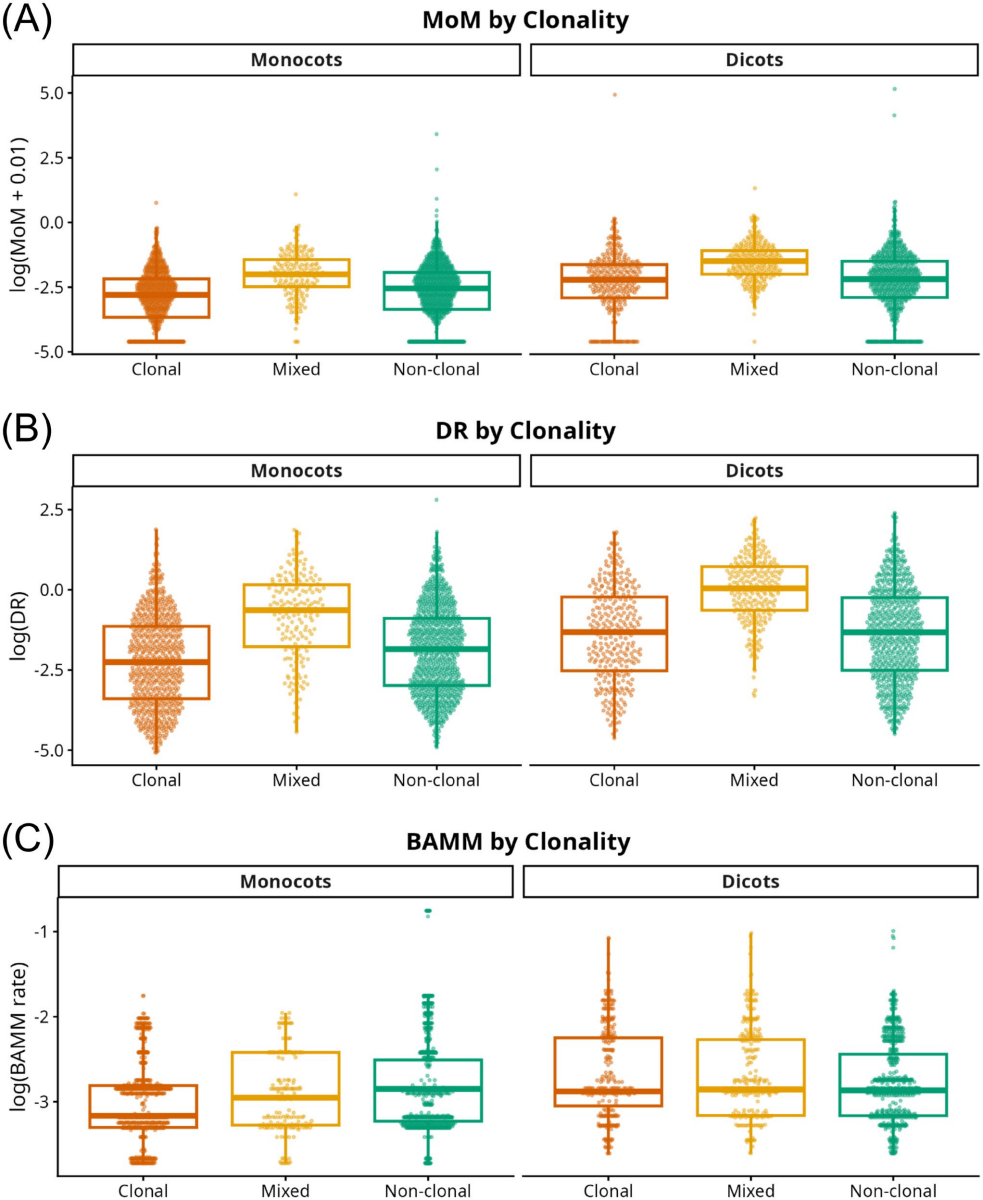
Comparison of MoM, DR and BAMM metrics across clonal groups in monocots and dicots. Boxplots show the distribution of log‐transformed values for three evolutionary metrics: (A) MoM (Mean of Means), (B) DR (Diversification Rate) and (C) BAMM (Bayesian Analysis of Macroevolutionary Mixtures) net diversification rate, separated by clade type (monocots vs. dicots) and clonality category (Clonal, Mixed, Non‐clonal). Mixed genera show higher diversification rates partly due to the effect of sampling effort; in our models, this was corrected for by including the sampling effort as a covariate. Each point represents an individual species. The boxes indicate the interquartile range (IQR), with the horizontal line showing the median. Whiskers extend to 1.5 × IQR, and points beyond whiskers are plotted as outliers.

The effects of clonality on diversification rates differed markedly between monocots and eudicots (Table 2). In monocots (*N* = 1700), linear models (LM) showed strong support for clonality effects (ΔAIC > 10 for DR and BAMM), with non‐clonal genera diversifying faster than clonal genera (*p* < 0.001). However, the support weakened after phylogenetic correction (PGLS: ΔAIC (clonality) ≤ 1.52; Table 2), indicating that the observed differences in diversification rates could be partly explained by shared evolutionary history among closely related genera rather than clonality alone. In eudicots (*N* = 1235), clonality effects were weaker and less consistent (Table 2) with only DR showing positive ΔAIC (clonality) values (LM: 5.95; PGLS: 3.08), and mixed genera rather than non‐clonal genera exhibiting elevated diversification rates compared to clonal genera (*β* (mixed) = 0.31, *p* < 0.01; Table 2). Non‐clonal eudicot genera did not differ significantly from clonal genera, and for BAMM, showed marginally lower diversification (*β* (non‐clonal) = −0.09, *p* < 0.05; Table 2). Interaction tests indicated that clonality effects on DR and BAMM differ significantly between monocots and eudicots (ΔAIC (interaction) > 10 in LM models), suggesting clade‐specific relationships between clonality and diversification. Across both monocots and eudicots, mixed genera tend to have higher MoM and DR values compared to clonal and non‐clonal genera. BAMM rates show less variation among clonality categories, with overlapping distributions across groups. Overall, these patterns suggest clonality is associated with differences in evolutionary dynamics (Figure 4).

**TABLE 2:** Effects of clonality on diversification rates in monocots and eudicots.

Model	Response	Clade	*N*	Intercept (Clonal)	Clonal vs. Mixed	Clonal vs. Non‐clonal	∆AIC (Clonality)	ΔAIC (Monocots vs. Dicot)	*λ*
LM	MoM	Monocots	1700	0.529***	0.315	0.229***	16.74	3.94	
	DR	Monocots	1700	0.852***	0.133	0.333***	28.54	11.45	
	BAMM	Monocots	1700	0.057***	0.012	0.169***	41.77	32.85	
PGLS	MoM	Monocots	1700	0.535***	0.148	0.143*	1.52	−2.04	0.45
	DR	Monocots	1700	0.857***	0.133	0.135	0.00	−3.92	0.74
	BAMM	Monocots	1700	0.005	−9e‐04	0.012	−1.84	−1.40	1.00
LM	MoM	Eudicots	1235	0.443***	0.099	−0.012	−2.71		
	DR	Eudicots	1235	0.648***	0.314*	−0.044	5.95		
	BAMM	Eudicots	1235	0.044*	−0.076	−0.086*	2.62		
PGLS	MoM	Eudicots	1235	0.476***	0.089	0.044	−3.24		0.44
	DR	Eudicots	1235	0.720***	0.308**	0.112	3.08		0.61
	BAMM	Eudicots	1235	0.009	0.001	−0.003	−3.65		1.00

*Note:* Results of linear models (LM) and phylogenetic generalized least squares (PGLS) examining the effect of clonality on log‐transformed diversification rates in monocots and eudicots. ΔAIC (clonality) = AIC of the model without clonality—AIC of the model including clonality (positive values indicate support for a clonality effect). ΔAIC (interaction) = AIC of the model without an interaction between clonality and monocot/dicot—AIC of the model including the interaction (positive values indicate that the effect of clonality differs between monocots and eudicots; it is listed only in the monocot table cells but refers to comparison of monocots and eudicots). *λ* = Pagel's *λ* (phylogenetic signal) for PGLS models. Significance: ***p < 0.001; **p < 0.01; *p < 0.05. See Table 1 for abbreviations.

## 4 Discussion

Our study shows that genera with only clonal species tend to exhibit slightly lower speciation rates compared to genera that contain species that reproduce only sexually. This supports our hypothesis that clonal mechanisms (lower recombination frequency and longer generation time) act to lower speciation and thus diversification rates. Differences in diversification rates between clonal and non‐clonal species could have a number of far‐reaching effects on plant evolutionary dynamics. However, two important issues should be mentioned here. First, although the overall effect of clonality on diversification is present, it is not particularly strong. Second, and more critically, the consistent effect of clonality on diversification across all three estimation methods is seen only in non‐phylogenetic models; if shared ancestry is controlled for using PGLS, it remains significant in MoM, is weaker in DR and disappears in BAMM. As we noted above, accounting for phylogenetic non‐independence provides us with an approximate test to what extent we may consider the results reflect parallel evolution or a simple effect of shared ancestry.

A key consideration in our analysis is the conceptual difference in the three diversification methods used here. MoM measures diversification only at the finest phylogenetic level and does not take into account processes taking place at the deeper‐than‐genus scales. In contrast, BAMM aims to detect significant shifts in diversification rates mostly in the deep parts of the phylogeny (Rabosky and Lovette 2008), with DR being intermediate between these two methods in accounting for processes at different phylogenetic scales. In addition, it should be noted that none of the three methods account for potential variability in the diversification process at the infrageneric level. For instance, recent radiations in groups like Lupinus (Drummond et al. 2012) or Ophrys (Breitkopf et al. 2015) may exhibit dramatic infrageneric interplay between diversification rates and body plan changes that in principle cannot be captured by our genus‐level analyses (see also Dimitrov et al. 2023 for the discussion of the issue).

The differences among the used diversification measures may explain the variation observed in our results in two different ways, one purely methodological and one biological. First, it is possible that deep‐time phylogenetically clustered variation of diversification rates captured by BAMM is more likely to get over‐compensated by the autocorrelation component of the used phylogenetic models. The same artefact, although weaker, may be present in DR, but is absent in MoM, because this measure is agnostic to the deeper than genus‐level processes. Second, it is possible that the effect of clonality is indeed phylogenetically scaled and would affect diversification rate at the finer phylogenetic levels rather than the deeper ones. This is consistent with the current understanding of the evolutionary flexibility of clonality (Herben and Klimešová 2020).

The explanation that clonality affects diversification mostly at within‐genus levels is also in line the observed high diversification rate in ‘mixed’ genera captured by our metrics. It might suggest that the transitions from clonal to non‐clonal strategies provide a temporary boost in diversification rates withing genera where such transition happens. However, the high diversification associated with ‘mixed’ genera might also easily result from a reversed causality effect where genera with higher diversity and thus longer within‐genus branch lengths are more likely to contain species that changed clonality status from the genus ancestral state, causing the genus to be ‘mixed’. We emphasize that these methodological and biological considerations around phylogenetic scaling cannot be fully addressed with the current study design, but could be explicitly disentangled by running diversification analyses at the species level and employing process‐based diversification models (e.g., Quintero et al. 2023; Beaulieu and O'Meara 2016). These approaches, however, require well‐resolved species‐level phylogenies and a more complete sampling of clonality than currently available.

The clonality‐diversification relationships differed between monocots and eudicots, likely due to the different body plans and evolutionary constraints operating in these two major angiosperm clades. Monocots generally do not have taproots (Givnish et al. 2018), which predispose them toward clonal growth strategies. However, the degree of clonality differs strongly among monocot plant species, partly depending on the type of clonal growth organ they possess (Howard et al. 2020; Klimešová et al. 2017). In monocots, there is a relationship between the type of clonal growth organ and genome size (Carta et al. 2020), suggesting that polyploidy and the huge differences in monoploid genome size known in monocots may be related both to clonal reproduction and diversification dynamics (Leitch and Leitch 2008; Vanneste et al. 2014).

In contrast, eudicots possess taproot systems and display a wider range of ecological strategies (Weigelt et al. 2021), which may account for their more variable rates of diversification and thus a weaker effect of clonality. The presence of woody growth forms in numerous eudicot lineages (Magallón and Sanderson 2001; Zanne et al. 2014) further diversifies their ecological strategies beyond simple clonal versus non‐clonal dichotomies. Recent studies have also shown that eudicots demonstrate higher rates of genome size variation and chromosomal evolution compared to monocots (Pellicer et al. 2018), which could contribute to their more complex diversification patterns.

The observed difference in diversification rates between clonal and non‐clonal species is likely to have implications for plant evolutionary dynamics and ecosystem functioning. While we cannot say with certainty that part of the effect is due to shared ancestry, it is likely that at lower phylogenetic levels, the link between clonality and diversification is independent of ancestry and represents a true functional relationship. Clonality is likely to be the ancestral state of the whole group (see Herben and Klimešová 2020), and our results suggest that shifts to sexual‐only reproductive mode might have sped up their diversification. The lower diversification rate in clonal lineages is perhaps too weak to act as an evolutionary ‘dead end’ (in contrast to fully asexual mode of reproduction, see e.g., Tucker et al. 2013; Neiman et al. 2014), but in some lineages, the widespread presence of clonality may be constraining their long‐term potential to generate new species.

Furthermore, the impact of clonality on evolutionary dynamics likely extends beyond simple speciation rates to influence extinction risk and lineage longevity. Clonal propagation allows for the very long persistence of successful genotypes (de Witte and Stöcklin 2010) and buffers populations against reproductive failure in the absence of mates or pollinators (Eckert et al. 1999; Vallejo‐Marín et al. 2010). Consequently, while clonal lineages may generate fewer species per unit of time, they may also experience lower background extinction rates, effectively trading high turnover for evolutionary persistence. This ‘longevity’ strategy could explain the widespread retention of clonality across the phylogeny despite its lower net diversification rates, as clonal taxa maintain occupancy of space through vegetative spread rather than rapid niche differentiation.

The observed patterns may reflect a fundamental trade‐off between immediate ecological success and long‐term adaptive potential. Clonal reproduction is often advantageous in habitats where environmental conditions limit seedling establishment (Eriksson 1992), yet reduced sexual recombination can limit the genetic variation necessary to adapt to rapid environmental changes (Otto 2009). Thus, the shift to a strictly sexual reproductive mode acts as a release from these constraints, accelerating the exploration of new adaptive landscapes and facilitating the accumulation of reproductive isolation barriers (Muller 1932; Hartfield and Keightley 2012).

Beyond evolutionary consequences, the clonality‐diversification relationship may also shape ecological patterns in habitats dominated by clonal species, potentially leading to slower changes in species composition over time. A slower pace of diversification in clonal species may suggest that habitats such as wetlands, alpine regions and other cold environments (Sosnová et al. 2010; Klimešová and Herben 2023; Chelli et al. 2023) may experience slower species turnover and also slower species accumulation over evolutionary time. The reduced pace of speciation in habitats dominated by clonal plants limits the generation of new species diversity, potentially slowing the process of adaptation. However, at the same time, once species are adapted to these environments, they could quickly reproduce and expand.

Finally, we should point out that a better understanding of the role of clonality is limited by the absence of representative and standardized clonal trait data across the angiosperm phylogeny. The use of the whole angiosperm genus level tree from Dimitrov et al. (2023) ameliorated the potential bias in diversification due to incomplete sampling of individual lineages, but incomplete sampling of clonal data may still affect the results. The species for which we have data are unevenly distributed across phylogeny, resulting in some lineages being overrepresented while others are underrepresented. Many of the analytical challenges encountered in this study could further be resolved by having large‐scale standardized clonal data using consistent definitions of clonality (Klimešová et al. 2019) and also better phylogenetic data. Developing comprehensive datasets on clonal status across species is an essential next step to fully reveal the evolutionary consequences of asexual reproduction in plants. Given the ecological prominence of clonal species in certain biomes, and the widespread occurrence of it in angiosperms, understanding their evolutionary trajectories is essential for predicting their responses to ongoing environmental changes and for informing conservation strategies.

## Author Contributions

S.K., T.H. and J.S. were involved in conceptualization and methodology. D.D. and Z.W. calculated BAMM diversification rates. J.K. provided clonality data. S.K. was involved in data assembly and curation, performed analyses, interpreted results and wrote the first draft of the manuscript. S.K., T.H. and J.S. also coordinated revisions and integrated feedback from all co‐authors. J.K., D.D., Z.W., J.S. and T.H. contributed substantially to the revisions. All authors approved the final version of the manuscript.

## Funding

This work was supported by Grantová Agentura České Republiky, 22‐10897S, 24‐12851O.

## Conflicts of Interest

The authors declare no conflicts of interest.

## Supporting information


**Data S1:** ele70423‐sup‐0001‐Supinfo1.csv.

**Table S1:** Sources and summary of datasets used in the analysis, listing the number of clonal and non‐clonal species included in each dataset.
**Table S2:** Sensitivity analysis of clonality effects on diversification rates accounting for phylogenetic uncertainty. Results from linear models (LM) and phylogenetic generalized least squares (PGLS) fitted to 1000 randomly sampled trees for assessing the effect of clonality on diversification rates. Mean parameter estimates, standard deviations (SD) and 95% confidence intervals (CI_95) summarize variation across trees. *R* ^2^ indicates model fit for LM. Lambda (*λ*) represents phylogenetic signal in PGLS residuals (0 = no signal, 1 = Brownian motion). The ‘Mixed’ and ‘Non‐clonal’ estimates represent the difference in rates compared to ‘Strictly Clonal’ genera (the reference level). A positive coefficient indicates that Mixed or Non‐clonal genera have higher diversification rates than Clonal genera. *λ* represents Pagel's lambda. Effects are considered significant if the 95% CI does not overlap zero.
**Figure S1:** Schematic overview of the methodological workflow used to analyse the relationship between clonality and diversification rates in flowering plants. Raw clonality data were harmonized taxonomically using World Flora Online, resulting in a dataset of 2997 genera. Phylogenetic data and diversification rates were sourced from Dimitrov et al. (2023). Three distinct diversification metrics were employed: Method of Moments (MoM) the DR metric and BAMM rates (extracted directly from the source study). Finally, the relationship between clonality and diversification rates was assessed using Phylogenetic Generalized Least Squares (PGLS) and Linear Models (LM).
**Figure S2:** Distribution of log‐transformed tip‐speciation rates (top row) and genus‐level net diversification rates (bottom row) for clonal (Aarsen 2008) and non‐clonal (0) species under crown age calibrations (260, 210 and 150 Ma). For each calibration, medians, interquartile ranges and overall distributions are shown for both groups. *p*‐values in each panel indicate the statistical comparison between clonal and non‐clonal categories. Open circles denote outliers.

## Data Availability

All data and R code supporting the analysis and results are freely available on Zenodo at: https://doi.org/10.5281/zenodo.19391481.

## References

[ele70423-bib-0001] Aarsen, L. W. 2008. “Death Without Sex: The ‘Problem of the Small’ and Selection for Reproductive Economy in Flowering Plants.” Evolutionary Ecology 22, no. 3: 279–298.

[ele70423-bib-0002] Beaulieu, J. M. , and B. C. O'Meara . 2016. “Detecting Hidden Diversification Shifts in Models of Trait‐Dependent Speciation and Extinction.” Systematic Biology 65, no. 4: 583–601. 27016728 10.1093/sysbio/syw022

[ele70423-bib-0003] Blum, M. G. B. , and O. François . 2006. “Which Random Processes Describe the Tree of Life? A Large‐Scale Study of Phylogenetic Tree Imbalance.” Systematic Biology 55, no. 4: 685–691. 16969944 10.1080/10635150600889625

[ele70423-bib-0004] Boedeltje, G. , W. A. Ozinga , and A. Prinzing . 2007. “The Trade‐Off Between Vegetative and Generative Reproduction Among Angiosperms Influences Regional Hydrochorous Propagule Pressure.” Global Ecology and Biogeography 17, no. 1: 50–58.

[ele70423-bib-0005] Breitkopf, H. , R. E. Onstein , D. Cafasso , P. M. Schlüter , and S. Cozzolino . 2015. “Multiple Shifts to Different Pollinators Fuelled Rapid Diversification in Sexually Deceptive Ophrys Orchids.” New Phytologist 207: 377–389. 25521237 10.1111/nph.13219

[ele70423-bib-0006] Carta, A. , G. Bedini , and L. Peruzzi . 2020. “A Deep Dive Into the Ancestral Chromosome Number and Genome Size of Flowering Plants.” New Phytologist 228: 1097–1106. 32421860 10.1111/nph.16668

[ele70423-bib-0007] Chelli, S. , J. Klimešová , J. L. Tsakalos , and G. Puglielli . 2023. “Clonality‐Related Traits Add Independent Specialization Axes to Herbs' Trait Strategies.” Preprint, bioRxiv, March 15. 10.1101/2023.03.15.532195.

[ele70423-bib-0008] Daru, B. H. , P. Karunarathne , and K. Schliep . 2020. “Phyloregion: R Package for Biogeographical Regionalization and Macroecology.” Methods in Ecology and Evolution 11, no. 11: 1483–1491.

[ele70423-bib-0009] de Witte, L. C. , and J. Stöcklin . 2010. “Longevity of Clonal Plants: Why It Matters and How to Measure It.” Annals of Botany 106, no. 6: 859–870. 20880935 10.1093/aob/mcq191 PMC2990663

[ele70423-bib-0010] Dimitrov, D. , X. Xu , X. Su , et al. 2023. “Diversification of Flowering Plants in Space and Time.” Nature Communications 14, no. 1: 7609. 10.1038/s41467-023-43396-8 PMC10665465 37993449

[ele70423-bib-0011] Drummond, C. S. , R. J. Eastwood , S. T. S. Miotto , and C. E. Hughes . 2012. “Multiple Continental Radiations and Correlates of Diversification in Lupinus (Leguminosae): Testing for Key Innovation With Incomplete Taxon Sampling.” Systematic Biology 61, no. 3: 443–460. 22228799 10.1093/sysbio/syr126 PMC3329764

[ele70423-bib-0012] Eckert, C. G. , M. E. Dorken , and S. A. Mitchell . 1999. “Loss of Sex in Clonal Populations of a Flowering Plant, *Decodon verticillatus* (Lythraceae).” Evolution 53, no. 4: 1079–1092. 28565532 10.1111/j.1558-5646.1999.tb04523.x

[ele70423-bib-0013] Ehrlén, J. , and K. Lehtilä . 2002. “How Perennial Are Perennial Plants?” Oikos 98, no. 2: 308–322.

[ele70423-bib-0014] Eriksson, O. 1992. “Evolution of Seed Dispersal and Recruitment in Clonal Plants.” Oikos 63: 439–448.

[ele70423-bib-0015] Eriksson, O. 1993. “Dynamics of Genets in Clonal Plants.” Trends in Ecology & Evolution 8, no. 9: 313–316. 21236180 10.1016/0169-5347(93)90237-J

[ele70423-bib-0016] Givnish, T. J. , A. Zuluaga , D. Spalink , et al. 2018. “Monocot Plastid Phylogenomics, Timeline, Net Rates of Species Diversification, the Power of Multi‐Gene Analyses, and a Functional Model for the Origin of Monocots.” American Journal of Botany 105, no. 11: 1888–1910. 30368769 10.1002/ajb2.1178

[ele70423-bib-0017] Hadany, L. , and S. P. Otto . 2016. “Condition‐Dependent Sex: Who Does It, When and Why?” Philosophical Transactions of the Royal Society, B: Biological Sciences 371, no. 1706: 20150539. 10.1098/rstb.2015.0539 PMC5031623 27619702

[ele70423-bib-0018] Hartfield, M. , and S. Glémin . 2016. “Limits to Adaptation in Partially Selfing Species.” Genetics 203, no. 2: 959–974. 27098913 10.1534/genetics.116.188821 PMC4896205

[ele70423-bib-0019] Hartfield, M. , and P. D. Keightley . 2012. “Current Hypotheses for the Evolution of Sex and Recombination.” Integrative Zoology 7, no. 2: 192–209. 22691203 10.1111/j.1749-4877.2012.00284.x

[ele70423-bib-0020] Herben, T. , and J. Klimešová . 2020. “Evolution of Clonal Growth Forms in Angiosperms.” New Phytologist 225, no. 2: 999–1010. 31505049 10.1111/nph.16188

[ele70423-bib-0021] Herben, T. , B. Šerá , and J. Klimešová . 2014. “Clonal Growth and Sexual Reproduction: Tradeoffs and Environmental Constraints.” Oikos 123, no. 11: 1325–1335.

[ele70423-bib-0022] Ho, L. S. T. , and C. Ané . 2014. “A Linear‐Time Algorithm for Gaussian Process Regression With Application to Phylogenetic Comparative Methods.” Computational Statistics and Data Analysis 56, no. 5: 988–996.

[ele70423-bib-0023] Howard, C. C. , J. B. Landis , J. M. Beaulieu , and N. Cellinese . 2020. “Geophytism in Monocots Leads to Higher Rates of Diversification.” New Phytologist 225, no. 2: 1023–1032. 31469440 10.1111/nph.16155

[ele70423-bib-0024] Ingram, T. , and D. L. Mahler . 2013. “SURFACE: Detecting Convergent Evolution From Comparative Data by Fitting Ornstein‐Uhlenbeck Models With Stepwise Akaike Information Criterion.” Methods in Ecology and Evolution 4: 416–425.

[ele70423-bib-0025] Jetz, W. , G. H. Thomas , J. B. Joy , K. Hartmann , and A. O. Mooers . 2012. “The Global Diversity of Birds in Space and Time.” Nature 491: 444–448. 23123857 10.1038/nature11631

[ele70423-bib-0026] Klimeš, L. , J. Klimešová , R. Hendriks , and J. Van Groenendael . 1997. “Clonal Plant Architecture: A Comparative Analysis of Form and Function.” In The Ecology and Evolution of Clonal Plants, vol. 1, 1–29. Backhuys Publishers.

[ele70423-bib-0027] Klimešová, J. , J. Danihelka , J. Chrtek , F. de Bello , and T. Herben . 2017. “CLO‐PLA: A Database of Clonal and Bud‐Bank Traits of the Central European Flora.” Ecology 98: 1179. 28122127 10.1002/ecy.1745

[ele70423-bib-0028] Klimešová, J. , and F. de Bello . 2009. “CLO‐PLA: The Database of Clonal and Bud Bank Traits of Central European Flora.” Journal of Vegetation Science 20, no. 3: 511–516.

[ele70423-bib-0029] Klimešová, J. , and T. Herben . 2023. “The Hidden Half of the Fine Root Differentiation in Herbs: Nonacquisitive Belowground Organs Determine Fine‐Root Traits.” Oikos 2023: e08794.

[ele70423-bib-0030] Klimešová, J. , J. Martínková , and G. Ottaviani . 2018. “Belowground Plant Functional Ecology: Towards an Integrated Perspective.” Functional Ecology 32, no. 9: 2115–2126.

[ele70423-bib-0031] Klimešová, J. , J. Martínková , J. G. Pausas , et al. 2019. “Handbook of Standardized Protocols for Collecting Plant Modularity Traits.” Perspectives in Plant Ecology, Evolution and Systematics 40: 125485.

[ele70423-bib-0032] Lanfear, R. , H. Kokko , and A. Eyre‐Walker . 2010. “Population Size and the Rate of Evolution.” Trends in Ecology & Evolution 25, no. 1: 43–51. 10.1016/j.tree.2013.09.009 24148292

[ele70423-bib-0033] Leitch, A. R. , and I. J. Leitch . 2008. “Genomic Plasticity and the Diversity of Polyploidy Plants.” Science 320: 481–483. 18436776 10.1126/science.1153585

[ele70423-bib-0034] Losos, J. B. , and R. E. Ricklefs . 2009. “Adaptation and Diversification on Islands.” Nature 457, no. 7231: 830–836. 19212401 10.1038/nature07893

[ele70423-bib-0035] Lynch, M. 1984. “The Limits to Life History Evolution in Daphnia.” Evolution 38, no. 3: 465–482. 28555980 10.1111/j.1558-5646.1984.tb00312.x

[ele70423-bib-0036] Magallón, S. , and M. J. Sanderson . 2001. “Absolute Diversification Rates in Angiosperm Clades.” Evolution 55: 1762–1780. 11681732 10.1111/j.0014-3820.2001.tb00826.x

[ele70423-bib-0037] Moore, B. R. , S. Höhna , M. R. May , B. Rannala , and J. P. Huelsenbeck . 2016. “Critically Evaluating the Theory and Performance of Bayesian Analysis of Macroevolutionary Mixtures.” Proceedings. National Academy of Sciences. United States of America 113: 9569–9574. 10.1073/pnas.1518659113 PMC5003228 27512038

[ele70423-bib-0038] Morlon, H. 2014. “Phylogenetic Approaches for Studying Diversification.” Ecology Letters 17, no. 4: 508–525. 24533923 10.1111/ele.12251

[ele70423-bib-0039] Muller, H. J. 1932. “Some Genetic Aspects of Sex.” American Naturalist 66, no. 703: 118–138.

[ele70423-bib-0040] Neiman, M. , S. Meirmans , and T. Schwander . 2014. “What Can Asexual Lineage Age Tell Us About the Maintenance of Sex?” Annals of the New York Academy of Sciences 1320, no. 1: 66–87. 10.1111/j.1749-6632.2009.04572.x 19566708

[ele70423-bib-0042] O'Meara, B. C. 2012. “Evolutionary Inferences From Phylogenies: A Review of Methods.” Annual Review of Ecology, Evolution, and Systematics 43: 227–247.

[ele70423-bib-0043] Orme, D. , R. Freckleton , G. Thomas , et al. 2018. “The Caper Package: Comparative Analysis of Phylogenetics and Evolution in R—R Package Version 1.01 2.10.” https://spout.ussg.indiana.edu/CRAN/web/packages/caper/vignettes/caper.pdf.

[ele70423-bib-0044] Otto, S. P. 2009. “The Evolutionary Enigma of Sex.” American Naturalist 174, no. Suppl 1: S1–S14. 10.1086/599084 19441962

[ele70423-bib-0045] Otto, S. P. , and T. Lenormand . 2002. “Resolving the Paradox of Sex and Recombination.” Nature Reviews. Genetics 3: 252–261. 10.1038/nrg761 11967550

[ele70423-bib-0046] Paradis, E. , and K. Schliep . 2019. “Ape 5.0: An Environment for Modern Phylogenetics and Evolutionary Analyses in R.” Bioinformatics 35, no. 3: 526–528. 30016406 10.1093/bioinformatics/bty633

[ele70423-bib-0047] Pausas, J. G. , B. B. Lamont , and N. J. Enright . 2018. “Unearthing Below‐Ground Bud Banks in Fire‐Prone Ecosystems.” New Phytologist 218, no. 2: 418–430. 10.1111/nph.14982 29334401

[ele70423-bib-0048] Pellicer, J. , O. Hidalgo , S. Dodsworth , and I. J. Leitch . 2018. “Genome Size Diversity and Its Impact on the Evolution of Land Plants.” Genes 9, no. 2: 88. 29443885 10.3390/genes9020088 PMC5852584

[ele70423-bib-0049] Quintero, I. , M. J. Landis , W. Jetz , and H. Morlon . 2023. “The Build‐Up of the Present‐Day Tropical Diversity of Tetrapods.” Proceedings of the National Academy of Sciences of the United States of America 120, no. 20: e2220672120. 37159475 10.1073/pnas.2220672120 PMC10194011

[ele70423-bib-0050] Rabosky, D. L. 2014. “Automatic Detection of Key Innovations, Rate Shifts, and Diversity‐Dependence on Phylogenetic Trees.” PLoS One 9, no. 2: e89543. 24586858 10.1371/journal.pone.0089543 PMC3935878

[ele70423-bib-0051] Rabosky, D. L. , J. Chang , P. O. Title , et al. 2018. “An Inverse Latitudinal Gradient in Speciation Rate for Marine Fishes.” Nature 559: 392–395. 29973726 10.1038/s41586-018-0273-1

[ele70423-bib-0052] Rabosky, D. L. , and E. E. Goldberg . 2015. “Model Inadequacy and Mistaken Inferences of Trait‐Dependent Speciation.” Systematic Biology 64: 340–355. 25601943 10.1093/sysbio/syu131

[ele70423-bib-0053] Rabosky, D. L. , and I. J. Lovette . 2008. “Explosive Evolutionary Radiations: Decreasing Speciation or Increasing Extinction Through Time?” Evolution 62, no. 8: 1866–1875. 18452577 10.1111/j.1558-5646.2008.00409.x

[ele70423-bib-0054] Rangel, T. F. , R. K. Colwell , G. R. Graves , K. Fučíková , C. Rahbek , and J. A. F. Diniz‐Filho . 2015. “Phylogenetic Uncertainty Revisited: Implications for Ecological and Evolutionary Studies.” Evolution 69: 1301–1312. 25800868 10.1111/evo.12644

[ele70423-bib-0055] Revell, L. J. 2012. “Phytools: An R Package for Phylogenetic Comparative Biology (And Other Things).” Methods in Ecology and Evolution 3: 217–223.

[ele70423-bib-0056] Revell, L. J. 2024. “Phytools 2.0: An Updated R Ecosystem for Phylogenetic Comparative Methods (And Other Things).” PeerJ 12: e16505. 38192598 10.7717/peerj.16505 PMC10773453

[ele70423-bib-0057] Ridder, G. I. , J. Smyčka , D. Storch , A. Ø. Mooers , and S. P. Otto . 2025. “Tip Rate Estimates Can Predict Future Diversification, but Are Unreliable and Context Dependent.” Preprint, bioRxiv, October 7. 10.1101/2025.10.06.680809.

[ele70423-bib-0058] Ronquist, F. , J. Kudlicka , V. Senderov , et al. 2021. “Universal Probabilistic Programming Offers a Powerful Approach to Statistical Phylogenetics.” Communications Biology 4: 244. 33627766 10.1038/s42003-021-01753-7 PMC7904853

[ele70423-bib-0059] Silvertown, J. 2008. “The Evolutionary Maintenance of Sexual Reproduction: Evidence From the Ecological Distribution of Asexual Reproduction in Clonal Plants.” International Journal of Plant Sciences 169, no. 1: 157–168.

[ele70423-bib-0060] Smyčka, J. , C. Roquet , J. Renaud , W. Thuiller , N. E. Zimmermann , and S. Lavergne . 2017. “Disentangling Drivers of Plant Endemism and Diversification in the European Alps—A Phylogenetic and Spatially Explicit Approach.” Perspectives in Plant Ecology, Evolution and Systematics 28: 1–10.

[ele70423-bib-0061] Smyčka, J. , A. Toszogyova , and D. Storch . 2023. “The Relationship Between Geographic Range Size and Rates of Species Diversification.” Nature Communications 14: 5559. 10.1038/s41467-023-41225-6 PMC10492861 37689787

[ele70423-bib-0062] Sosnová, M. , R. van Diggelen , and J. Klimešová . 2010. “Distribution of Clonal Growth Forms in Wetlands.” Aquatic Botany 92, no. 1: 33–39.

[ele70423-bib-0063] Title, P. O. , and D. L. Rabosky . 2019. “Tip Rates, Phylogenies and Diversification: What Are We Estimating, and How Good Are the Estimates?” Methods in Ecology and Evolution 10: 821–834.

[ele70423-bib-0064] Tucker, A. E. , M. S. Ackerman , B. D. Eads , S. Xu , and M. Lynch . 2013. “Population‐ Genomic Insights Into the Evolutionary Origin and Fate of Obligately Asexual *Daphnia pulex* .” Proceedings. National Academy of Sciences. United States of America 110, no. 39: 15740–15745. 10.1073/pnas.1313388110 PMC3785735 23959868

[ele70423-bib-0065] Ülgen, C. , and Ç. Tavşanoğlu . 2024. “A Taxonomic Snapshot of Belowground Organs in Plants of Anatolian Steppes.” Folia Geobotanica 58, no. 3–4: 231–243.

[ele70423-bib-0066] Vallejo‐Marín, M. , M. E. Dorken , and S. C. Barrett . 2010. “The Ecological and Evolutionary Consequences of Clonality for Plant Mating.” Annual Review of Ecology, Evolution, and Systematics 41: 193–213.

[ele70423-bib-0067] Vanneste, K. , Y. Van de Peer , and S. Maere . 2014. “Inference of Genome Duplications From Age Distributions.” Molecular Biology and Evolution 30, no. 1: 177–190. 10.1093/molbev/mss214 22936721

[ele70423-bib-0068] Weigelt, A. , L. Mommer , K. Andraczek , et al. 2021. “An Integrated Framework of Plant Form and Function.” New Phytologist 232: 1142–1158. 10.1111/nph.17590 34197626

[ele70423-bib-0069] World Flora Online (WFO) . 2023. “World Flora Online.” http://www.worldfloraonline.org.

[ele70423-bib-0070] Xue, J. Z. , Z. Deng , P. Huang , et al. 2016. “Belowground Rhizomes in Paleosols: The Hidden Half of an Early Devonian Vascular Plant.” Proceedings of the National Academy of Sciences of the United States of America 113: 9451–9456. 27503883 10.1073/pnas.1605051113 PMC5003246

[ele70423-bib-0071] Zanne, A. E. , D. C. Tank , W. K. Cornwell , et al. 2014. “Three Keys to the Radiation of Angiosperms Into Freezing Environments.” Nature 506, no. 7486: 89–92. 24362564 10.1038/nature12872

[ele70423-bib-0072] Zhang, H. , S. P. Bonser , S.‐C. Chen , T. Hitchcock , and A. T. Moles . 2017. “Is the Proportion of Clonal Species Higher at Higher Latitudes in Australia?” Austral Ecology 42, no. 3: 279–287.

