# Supplementary material for: Comparative Analysis of Diversification Rates in Clonal and Non‐Clonal Flowering Plants
Source: Ecol Lett. 2026 Jun 7;29(6):e70423. doi: 10.1111/ele.70423 (PMC13243725; doi:10.1111/ele.70423)
Supplement: Supplementary file 2 — Table S1: Sources and summary of datasets used in the analysis, listing the number of clonal and non‐clonal species included in each dataset. Table S2: Sensitivity analysis of clonality effects on diversification rates accounting for phylogenetic uncertainty. Results from linear models (LM) and phylogenetic generalized least squares (PGLS) fitted to 1000 randomly sampled trees for assessing the effect of clonality on diversification rates. Mean parameter estimates, standard deviations (SD) and 95% confidence intervals (CI_95) summarize variation across trees. R 2 indicates model fit for LM. Lambda (λ) represents phylogenetic signal in PGLS residuals (0 = no signal, 1 = Brownian motion). The ‘Mixed’ and ‘Non‐clonal’ estimates represent the difference in rates compared to ‘Strictly Clonal’ genera (the reference level). A positive coefficient indicates that Mixed or Non‐clonal genera have higher diversification rates than Clonal genera. λ represents Pagel's lambda. Effects are considered significant if the 95% CI does not overlap zero. Figure S1: Schematic overview of the methodological workflow used to analyse the relationship between clonality and diversification rates in flowering plants. Raw clonality data were harmonized taxonomically using World Flora Online, resulting in a dataset of 2997 genera. Phylogenetic data and diversification rates were sourced from Dimitrov et al. (2023). Three distinct diversification metrics were employed: Method of Moments (MoM) the DR metric and BAMM rates (extracted directly from the source study). Finally, the relationship between clonality and diversification rates was assessed using Phylogenetic Generalized Least Squares (PGLS) and Linear Models (LM). Figure S2: Distribution of log‐transformed tip‐speciation rates (top row) and genus‐level net diversification rates (bottom row) for clonal (Aarsen 2008) and non‐clonal (0) species under crown age calibrations (260, 210 and 150 Ma). For each calibration, medians, interquartile rang [file ELE-29-0-s001.docx]

**Supplementary Information for**

**Title: Comparative Analysis of Diversification Rates in Clonal and Non-Clonal Flowering Plants**

Sonia Kadyan¹, Jitka Klimešová^1,2^, Dimitar Dimitrov³, Zhiheng Wang⁴, Jan Smyčka^5,6,7^, Tomáš Herben^1,2^

^1^ Department of Botany, Faculty of Science, Charles University, Praha, Czech Republic

^2^ Institute of Botany, Czech Academy of Sciences, Průhonice, Czech Republic

^3^ Department of Natural History, University Museum of Bergen, University of Bergen, Norway

^4^ Institute of Ecology and State Key Laboratory for Vegetation Structure, Function and Construction, College of Urban and Environmental Sciences, Peking University, Beijing, China

^5^ Center for Theoretical Studies, Charles University, Praha, Czech Republic

^6^ Biological Sciences, Simon Fraser University, Burnaby, BC, Canada

^7^ Biodiversity Research Centre, University of British Columbia, Vancouver, BC, Canada

Correspondence: Sonia Kadyan. Email: [soniakadyan1996@gmail.com](mailto:soniakadyan1996@gmail.com); Tel: +420775844189

**Contents**

**1 Appendix S1: Supplementary table**

**2 Appendix S2: Supplementary figures**

**1 Appendix S1: Supplementary table**

Table S1 summarizes the datasets compiled and analyzed in this study, including the number of clonal and non-clonal species contributed by each source. Data were obtained from both published studies and unpublished contributions, encompassing a wide range of taxa and geographic regions.
Across all datasets, a total of **8,359 clonal species** and **10,238 non-clonal species** were included, representing comprehensive coverage of plant functional diversity relevant to clonal growth strategies. These datasets formed the basis for taxonomic harmonization, data cleaning, and subsequent comparative and phylogenetic analyses.

**Table S1. Sources and summary of datasets used in the analysis, listing the number of clonal and non-clonal species included in each dataset.**

| Dataset source | No. of Clonal Species | No. of Non-clonal Species | Total No. of Species |
| --- | --- | --- | --- |
| CLO-PLA | 1546 | 1329 | 2875 |
| Jitka Klimešová Unpublished Data | 1011 | 929 | 1941 |
| Zhang et al. (2017) | 471 | 3245 | 4116 |
| Howard et al. (2020) | 3092 | 4462 | 7554 |
| Pausas et al. (2018) | 599 | 180 | 779 |
| Ülgen and Çağatay (2024) | 640 | 93 | 736 |

**Data File S1: Species-level clonality dataset**

The complete species-level clonality dataset is provided in the supporting information (Data_S1_clonality_species.csv). This data set formed the basis for all genus-level aggregations and phylogenetic analyses reported in this study. Species names were taxonomically harmonized using the World Flora Online taxonomic backbone (WFO, 2024), and duplicate entries were resolved by cross-referencing data sources. This file was used to generate genus-level clonality classifications and was merged with phylogenetic data from Dimitrov et al. (2023) for all downstream analysis.

**Supplementary table S2: Sensitivity to Phylogenetic Uncertainty**

To assess the impact of uncertainty in DR metric caused by species-level augmentation of Dimitrov et al. 2023 genus-level phylogeny, we conducted a sensitivity analysis by fitting Linear Models (LM) and Phylogenetic Generalized Least Squares (PGLS) models across 1,000 phylogenetic trees with randomly generated infrageneric branching. The parameter estimates demonstrated remarkable stability across the different tree topologies and branch lengths, with negligible variation in model coefficients (Appendix, Table S2; Fig. S2). The 95% confidence intervals for the effects of reproductive mode and sampling effort were very narrow and did not overlap zero in any of the summarized metrics (Appendix, Table S2). This confirms that the statistical significance of the predictors remains robust across phylogenetic uncertainty, yielding consistent results for the vast majority of the diversity augmented trees.

| **Model** | **Parameter** | **Mean** | **SD** | **CI_95** |
| --- | --- | --- | --- | --- |
| **LM** | Sampling effort | 0.208 | 0.001 | [0.206, 0.211] |
|  | Mixed vs Clonal | 0.172 | 0.004 | [0.164, 0.181] |
|  | Non-clonal vs Clonal | 0.154 | 0.004 | [0.147, 0.161] |
|  | R² | 0.047 | 0.001 | [0.046, 0.048] |
|  | Δ AIC (clonality) | 9.1 | 0.6 | [7.9, 10.3] |
| **PGLS** | Sampling effort | 0.273 | 0.002 | [0.27, 0.276] |
|  | Mixed vs Clonal | -0.103 | 0.006 | [-0.115, -0.092] |
|  | Non-clonal vs Clonal | 0.024 | 0.005 | [0.014, 0.033] |
|  | Lambda (λ) | 0.707 | 0.005 | [0.697, 0.717] |
|  | ΔAIC (clonality) | 1.37 | 0.42 | [0.53, 2.16] |

**Table S2. Sensitivity analysis of clonality effects on diversification rates accounting for phylogenetic uncertainty. Results from linear models (LM) and phylogenetic generalized least squares (PGLS) fitted to 1,000 randomly sampled trees for assessing the effect of clonality on diversification rates. Mean parameter estimates, standard deviations (SD), and 95% confidence intervals (CI_95) summarize variation across trees. R² indicates model fit for LM. Lambda (λ) represents phylogenetic signal in PGLS residuals (0 = no signal, 1 = Brownian motion). The "Mixed" and "Non-clonal" estimates represent the difference in rates compared to "Strictly Clonal" genera (the reference level). A positive coefficient indicates that Mixed or Non-clonal genera have higher diversification rates than Clonal genera. λ represents Pagel’s lambda. Effects are considered significant if the 95% CI does not overlap zero.**

**2 Appendix S1: Supplementary figures**

**Supplementary figure S1**

Figure S1 illustrates the complete workflow used for data processing and analysis in this study. The flowchart details each step in order, starting with data collection, where species occurrence records and related metadata were gathered from multiple databases. Taxonomic standardization was carried out to unify species names and resolve synonyms. The next steps involved filtering and cleaning the data to remove incomplete or inconsistent entries, ensuring data quality. The curated dataset was then combined with phylogenetic information to incorporate evolutionary relationships among taxa. Final analyses were performed at the genus and species levels, including statistical tests to evaluate diversification patterns.


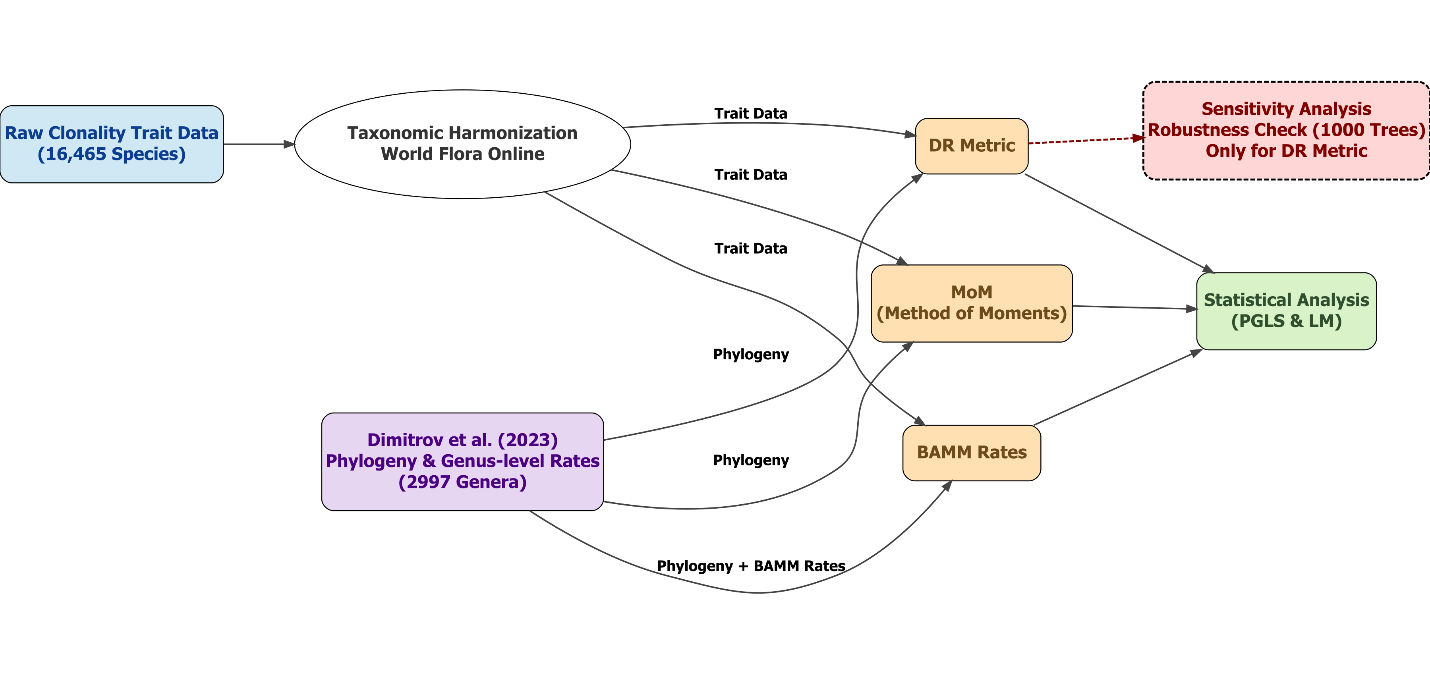


**Figure S1. Schematic overview of the methodological workflow used to analyse the relationship between clonality and diversification rates in flowering plants.**Raw clonality data were harmonized taxonomically using World Flora Online, resulting in a dataset of 2,997 genera. Phylogenetic data and diversification rates were sourced from Dimitrov et al. (2023). Three distinct diversification metrics were employed: Method of Moments (MoM) the DR metric and BAMM rates (extracted directly from the source study). Finally, the relationship between clonality and diversification rates was assessed using Phylogenetic Generalized Least Squares (PGLS) and Linear Models (LM).

**Supplementary figure S2**

Beeswarm plots display the distribution of log-transformed genu-level speciation rates (top row) and genus-level net diversification rates (bottom row) for clonal, mixed, and non-clonal genera under three crown age calibrations (260, 210, and 150 Ma). Each point represents an individual observation, with horizontal spread reflecting data density. Overlaid horizontal bars indicate median values and interquartile ranges. P-values in each panel denote statistical comparisons among groups.

**
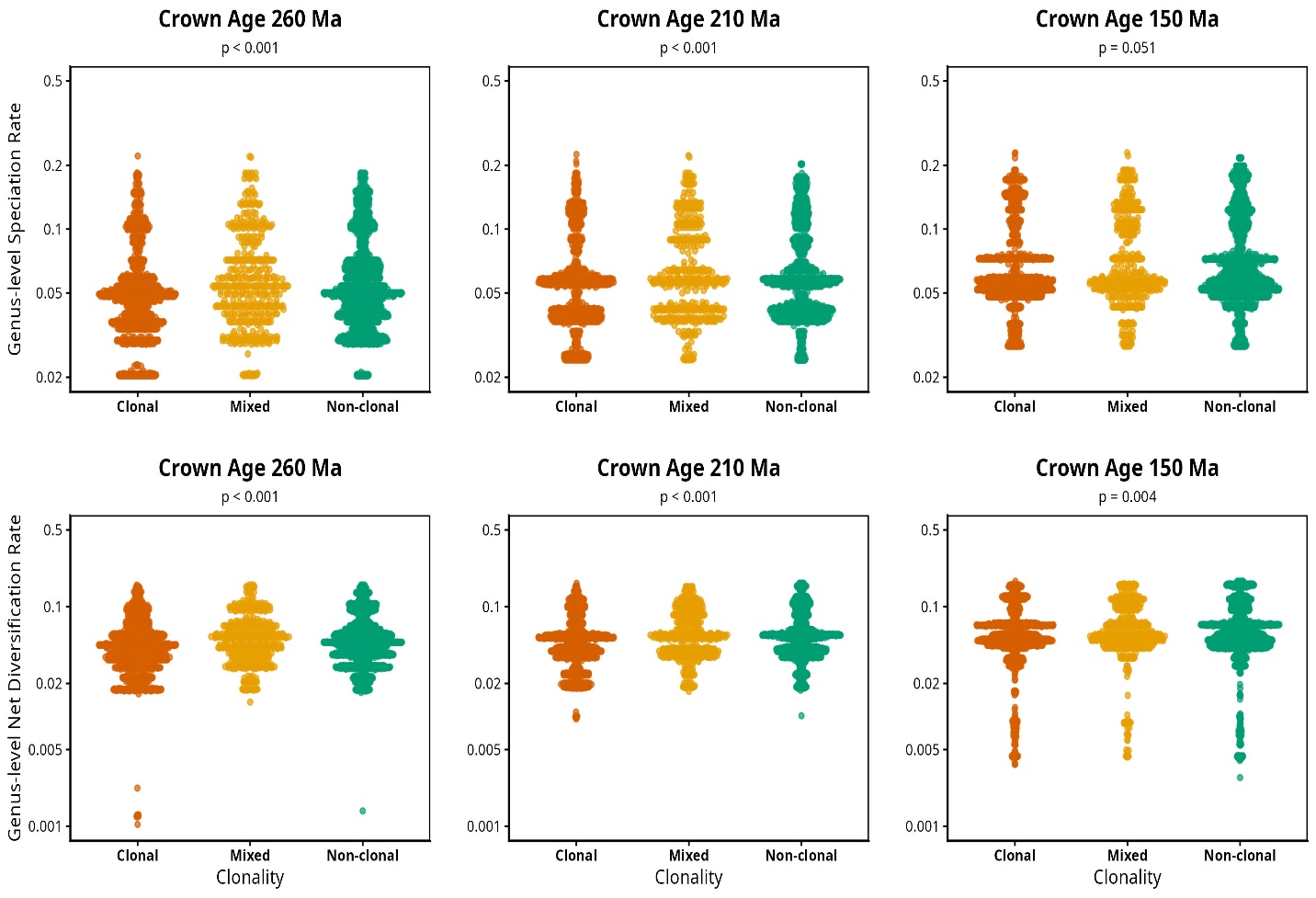
**

**Figure S2. Distribution of log-transformed tip-speciation rates (top row) and genus-level net diversification rates (bottom row) for clonal (1) and non-clonal (0) species under crown age calibrations (260, 210, and 150 Ma). For each calibration, medians, interquartile ranges, and overall distributions are shown for both groups. P-values in each panel indicate the statistical comparison between clonal and non-clonal categories. Open circles denote outliers.**

**References for Table S1**

1. **Klimešová, J., Danihelka, J., Chrtek, J., de Bello, F., & Herben, T. (2017).** CLO-PLA: a database of clonal and bud-bank traits of the Central European flora. *Ecology* (Vol. 98, p. 1179). https://doi.org/10.1002/ecy.1745
2. **Zhang, L., Zhang, Z., & Zhang, Q. (2017**). Is the proportion of clonal species higher at higher latitudes in Australia? *Austral Ecology, 42*(3), 279-287. DOI: 10.1111/aec.12536.
3. **Howard, C. C., Landis, J. B., Beaulieu, J. M., & Cellinese, N. (2020).** Geophytism in monocots leads to higher rates of diversification. *New Phytol.*, *225*(2), 1023-1032. DOI: 10.1111/nph.16155
4. **Pausas, J. G., Lamont, B. B., & Enright, N. J. (2018).** Unearthing below-ground bud banks in fire-prone ecosystems. *New Phytol*, 218(2), 418–430. DOI: 10.1111/nph.14982.
5. **Ülgen, C., & Tavşanoğlu, Ç. (2024).** A taxonomic snapshot of belowground organs in plants of Anatolian steppes. *Folia Geobotanica*, *58*(3–4), 231–243. https://doi.org/10.1007/s12224-024-09442-z.
6. **Dimitrov D., et., al.** (2023). Diversification of flowering plants in space and time. Nature Communications, 14, 7609. https://doi.org/10.1038/s41467-023-43396-8.
